# Investigating the Impact of Fasting and Refeeding on Blood Biochemical Indicators and Transcriptional Profiles in the Hypothalamus and Subcutaneous Adipose Tissue in Geese

**DOI:** 10.3390/ani14182746

**Published:** 2024-09-23

**Authors:** Yi Liu, Xianze Wang, Guangquan Li, Shufang Chen, Huiyan Jia, Jiuli Dai, Daqian He

**Affiliations:** 1 Shanghai Academy of Agricultural Sciences, Shanghai 201106, China; liuyi20031194@163.com (Y.L.); wxz213187058389@163.com (X.W.); lgqdx123@126.com (G.L.); 2 Ningbo Academy of Agricultural Sciences, Ningbo 315101, China; 13606780161@163.com (S.C.); jhynku@163.com (H.J.); 13858357201@163.com (J.D.)

**Keywords:** transcriptome analysis, goose, hypothalamus, subcutaneous adipose tissue, gene expression

## Abstract

**Simple Summary:**

This study explores how geese respond to fasting and subsequent refeeding by analyzing changes in blood chemistry and gene activity in the hypothalamus and subcutaneous adipose tissues. After 24 h of fasting, geese’s blood levels of key substances such as fatty acids, glucagon, triglycerides, leptin, and insulin changed noticeably. The research also identified specific genes in the hypothalamus and subcutaneous adipose tissue that are influenced by fasting, providing insight into how geese regulate energy and maintain metabolic balance in response to changes in food intake.

**Abstract:**

Fasting and refeeding systems can cause significant short-term fluctuations in nutrient and energy levels, triggering adaptive physiological responses in animals. This study examines the effects of fasting and refeeding on blood biochemical indicators and transcriptional profiles in the hypothalamus and subcutaneous adipose tissue of geese. Biochemical assays reveal that fasting significantly increases levels of free fatty acids and glucagon, while reducing concentrations of triglycerides, leptin, and insulin. Transcriptomic analyses identify a complex transcriptional response in both the hypothalamus and subcutaneous adipose tissue, affecting several metabolic pathways and key genes associated with feed intake and energy metabolism. In subcutaneous adipose tissue, fasting downregulates genes involved in fatty acid synthesis (*LPL*, *SCD*, and *ACSL1*) and upregulates *PLIN2*, a gene promoting lipid droplet degradation. Fasting affects a variety of metabolic pathways and critical genes in the hypothalamus, including Apelin, insulin, and mTOR signaling pathways. After fasting, the mRNA expression of *NOG*, *GABRD*, and *IGFBP-1* genes in the hypothalamus are significantly upregulated, while proopiomelanocortin (*POMC*) gene expression is markedly downregulated. This study highlights the intricate biological responses to nutritional changes in geese, which adds to our understanding of energy balance and metabolic regulation in avian species.

## 1. Introduction

The consumption of waterfowl meat is a significant source of poultry in Asia, particularly in China, where this trend is steadily increasing (Food and Agriculture Organization of the United Nations (FAO) database http://faostat.fao.org) (accessed on 12 May 2024). In fact, China accounts for over 90% of global goose meat production, making it the world’s largest producer [1]. Ducks [2] and geese [3] have higher fat content in their carcasses than other poultry, with significant fat accumulation in the abdominal region and subcutaneous tissue. Subcutaneous fat is economically important because it improves the flavor and texture of meat and is closely related to meat quality and feed conversion rate. A new strain of Fat Pekin Duck (FPD) has been selectively bred specifically for roast duck production, with subcutaneous fat ratio and skin thickness as key selection criteria [4]. Adipose tissue distribution in different parts of poultry varies metabolically [5,6,7], and it is influenced by feed intake and genetic type. Overfeeding increases fat deposition, and different genotypes show significant variation in fat storage in subcutaneous adipose tissue and the liver [8,9]. Previous research on lipid metabolism in geese focused on quantifying plasma metabolites and studying the activities of key enzymes involved in hepatic lipid synthesis, storage, and secretion to better understand the mechanisms underlying hepatic steatosis development [10,11,12]. However, there is limited knowledge regarding the mechanisms and physiological properties of lipid metabolism in extrahepatic tissues, particularly subcutaneous fat deposition, in geese. The previous discussion highlights the significance of feed intake in body fat accumulation in waterfowl and its impact on adipose tissue distribution. Additionally, feed intake plays a crucial role in the efficiency and cost-effectiveness of waterfowl production.

In animals, the central nervous system (CNS) regulates food intake, hunger, satiety, adipose tissue storage, and energy homeostasis [13,14]. A key component of the CNS is the hypothalamus, which directly influences feed intake [15,16] and energy balance [17] in birds. Previous studies have demonstrated the role of alpha-melanocyte stimulating hormone (α-MSH) in regulating food intake through the central melanocortin system in chicks [18] and quails [19]. The hypothalamic signaling pathway is associated with energy balance via appetite-regulating factors such as neuropeptide Y (NPY) and melanocortin, influencing the physiology of subcutaneous fat in quail [20,21]. However, the relationship between the hypothalamus and the regulation of feed intake and subcutaneous fat deposition in geese remains poorly understood. Understanding the regulatory mechanisms of feed intake in geese, as well as the physiological characteristics of subcutaneous fat deposition is crucial for accurately breeding lean and obese geese. This knowledge is also essential for developing new approaches to enhance feed conversion efficiency and meat quality in geese.

Fasting and refeeding systems can cause significant short-term fluctuations in nutrient and energy levels, triggering adaptive physiological responses in animals. These experimental approaches are frequently used to investigate the molecular mechanisms by which nutrient and energy metabolism affect physiological function and disease progression in animals [22,23]. Previous studies on hepatic lipogenesis and metabolism during fasting and refeeding have identified specific genes and metabolic pathways that regulate energy metabolism in poultry. For example, fasting in chickens activates the hepatic adenosine monophosphate-activated protein kinase (AMPK) pathway, which suppresses the expression of genes such as glycogen synthase (GS), fatty acid synthase (FAS), and sterol regulatory element-binding proteins (SREBP-1) [24]. The thyroid hormone responsive spot 14 A (THRSPA) transcriptional regulator has emerged as the pivotal driver of lipogenesis and thermogenesis in hatchling chicks during both fed and re-fed states [25]. Fasting primarily affects hepatic metabolism, particularly lipid metabolism, while the peroxisome proliferator-activated receptor (PPAR) signaling pathway potentially regulates these lipid metabolic processes [26]. Changes in mRNA levels of appetite-related factors in the hypothalamus differ following fasting and refeeding, suggesting a potential link between neuropeptide Y receptor type 5 (NPY5R) and physiological changes in subcutaneous adipose tissue in 7-day-old Japanese quail [20,21]. These studies provide valuable insights into the mechanisms of lipid metabolism in poultry under fasting and refeeding protocols. Fasting and refeeding can help us better understand the effects of nutritional and energy changes on feeding behavior, hunger, satiety, and the molecular mechanisms of lipid metabolism in geese.

The period of 4–8 weeks during early growth is critical for rapid body weight gain and subcutaneous fat deposition in geese [27]. As a result, we examined serum biomarkers, subcutaneous adipose tissue sections, and the transcriptome of the hypothalamus and subcutaneous adipose tissue at 42 days post-hatch in geese during fasting and subsequent refeeding periods. These investigations provide valuable insights into the lipid metabolism characteristics found in subcutaneous adipose tissue in early-stage geese and examine the role of the hypothalamus in this physiological process. This study contributes to a comprehensive understanding of the genes and gene interaction networks that regulate lipid biological processes.

## 2. Materials and Methods

### 2.1. Animal Ethics

We adhere to the 2017 Regulations of The State Council of the People’s Republic of China on the Management of Experimental Animals. This study was authorized by the Animal Ethics Committee of the Shanghai Academy of Agricultural Sciences (Shanghai, China) under approval number SAASPZ0522046 in accordance with ethical guidelines for animal research.

### 2.2. Animals and Sample Collection

The experiment was conducted at the Zhuanghang Research Farm, affiliated with the Shanghai Academy of Agricultural Sciences in Shanghai, China. A total of 108 one-day-old male Zhedong White Geese, with similar weights and incubated in the same batch at the Zhuanghang Research Farm, were randomly divided into three groups. Each group had six replicates, with six geese per replicate. The geese were randomly assigned to 6 m^2^ net beds (3 × 2 m) for indoor rearing at a stocking density of 1 m^2^ per head. The flooring is a specially designed PVC manure leakage board for geese, with a hole diameter of 12 mm × 12 mm. The flooring is elevated 60 cm above the ground and is surrounded by metal wire mesh with a plastic coating (each mesh size is 1 cm × 1 cm). Each pen is equipped with a plastic feeder, 30 cm in height and 35 cm in base diameter, and four nipple drinkers. These facilities can be adjusted according to the height of the geese to ensure that all geese have easy access to food and water. During the initial two-week feeding period, the geese were exposed to constant 24 h illumination with a light intensity of 4 to 5 W/m^2^. The temperature was maintained between 28 and 32 °C, and the ambient relative humidity was kept between 55 and 65%. From 15 to 42 days of age, the geese experienced a 12 h light–dark cycle, with lights on from 08:00 to 20:00. The light intensity was maintained at 4 to 5 W/m^2^. The ambient temperature was controlled within the range of 23 to 25 °C, and the relative humidity was maintained between 50 and 60%. The diets were formulated based on the National Research Council (NRC) (1994) standards and adjusted according to the specific nutritional requirements of geese at different developmental stages, ensuring consistent primary nutrient levels across all experimental groups (Table 1). Throughout the experiment, all geese were kept under consistent light and temperature conditions, with free access to water and feed. Fresh feed was provided twice daily to ensure that the feeders and water lines were always filled with sufficient feed and water. At 42 days of age, the first group continued its previous feeding regimen until the completion of fasting and refeeding periods for the other two groups. The second and third groups commenced fasting (the second group from 11:00 a.m. and the third group from 8:00 a.m.), while having unrestricted access to water during the fasting period. The third group underwent a refeeding phase lasting for 3 h after completing a 24 h fast.

At 43 days of age (11:00 a.m.), one representative goose with an average body weight was selected from each replicate, resulting in a total of 18 samples with an average weight of 1898.52 ± 84.22 g (six per group). Blood samples were collected from the subwing veins and stored at 4 °C for 20 min. The samples were then centrifuged at 3500× *g* for 15 min at 4 °C to obtain serum, which was then stored at −20 °C for compositional analysis. Eighteen geese were euthanized by carbon dioxide inhalation followed by cervical dislocation, performed by trained personnel. After euthanasia, we collected abdominal subcutaneous adipose tissue samples measuring 1 cm × 1 cm from the midline, 3 cm below the cloaca, and immediately flash-frozen in liquid nitrogen for RNA extraction and stored at −80 °C. Additionally, we collected hypothalamic arcuate nucleus (ARC) tissue samples measuring 0.5 cm × 0.5 cm from the medial basal hypothalamus near the median eminence, with one sample collected from the same position on both the left and right sides. One hypothalamus sample was fixed with in situ hybridization fixative (4% paraformaldehyde without RNA enzymes), while the remaining samples were immediately flash-frozen in liquid nitrogen for RNA extraction and stored at −80 °C.

### 2.3. Serum Biochemical Analysis

The levels of serum lipid biochemical indicators, including free fatty acids (FFAs), triglyceride (TG), total cholesterol (TC), high-density lipoprotein (HDL), low-density lipoprotein (LDL), and very low-density lipoprotein (VLDL), were measured using commercially available kits from Shenzhen Mindray Bio-Medical Electronics Co., Ltd. in Shenzhen, China. The analysis was carried out on an automated biochemical analyzer BS-200 (Mindray, Shenzhen, China), following the manufacturer’s instructions. The levels of serum appetite-related factors, including insulin, glucagon, leptin, and adiponectin, were quantified using enzyme-linked immunosorbent assay (ELISA) kits obtained from Shanghai Biotechnology Co., Ltd. (Shanghai, China). These assays were performed according to the manufacturer’s protocols for goose samples and analyzed using a multifunctional enzyme labeling instrument (infnite 200, TECAN, Mannedorf, Switzerland).

### 2.4. Total RNA Isolation and Transcriptome Sequencing

Total RNA was isolated from the hypothalamus and abdominal subcutaneous adipose tissue samples using Trizol Reagent (Invitrogen Life Technologies, Waltham, MA, USA) following the manufacturer’s protocol. The concentration and quality of the RNA were assessed using a NanoDrop spectrophotometer (Thermo Fisher Scientific Inc., Waltham, MA, USA), and its integrity was determined using agarose gel electrophoresis. The GS-FLX+ platform was used to perform transcriptome sequencing. A cDNA library was prepared from 3 µg of high-quality total RNA using Illumina’s NEBNext Ultra II RNA Library Prep Kit (New England Biolabs Inc.; Ipswich, MA, USA) following established protocols. The cDNA libraries underwent shearing, purification, end blunting, and ligation with adapters optimized specifically for Illumina sequencing. The AMPure XP system (Beckman Coulter, Beverly, CA, USA) was used to purify the library fragments, enriching cDNA fragments primarily ranging from 400–500 bp in length. Subsequently, Illumina PCR primers were employed to amplify DNA fragments linked to splice molecules at both ends through 15 cycles of PCR. The PCR products were further purified using the AMPure XP system and quantified using the Agilent 2100 Bioanalyzer (Agilent, Santa Clara, CA, USA). Finally, the libraries were sequenced on the NovaSeq 6000 platform (Illumina, San Diego, CA, USA) by Shanghai Personal Biotechnology Co., Ltd., Shanghai, China, generating 125–150 bp paired-end reads.

### 2.5. Transcriptome Analysis

To ensure high-quality sequencing, we employed the fastp software (version 0.22.0) to remove adapter sequences and low-quality reads from the raw data. The cleaned reads were then aligned to the reference genome from the official genome database website using HISAT2 (version 2.1.0) [28]. Gene expression levels were quantified using HTSeq (version 0.9.1) [29], which provided Read Counts per gene. These counts were normalized using the FPKM method to standardize expression levels. Differential gene expression (DGE) analysis was performed using DESeq2 (version 1.38.3) [30], applying criteria of an absolute log2FoldChange greater than 1 and *p*-value of less than 0.05 for significance. Additionally, we performed bi-directional clustering analysis on all differentially expressed genes using the R package Pheatmap (version 1.0.12) [31]. For gene ontology (GO) and Kyoto Encyclopedia of Genes and Genomes (KEGG) pathway enrichment analyses, we utilized the topGO (version 2.50.0) [32] and ClusterProfiler (version 4.6.0) [33] software, respectively, with a significance threshold of a *p*-value less than 0.05. These analyses facilitated the identification of significantly enriched primary biological functions and pathways among the differentially expressed genes.

### 2.6. Validation of DGE Results Using qRT-PCR

To validate the expression of selected genes identified through transcriptome analysis, we chose eight candidate genes that showed significant differential expression. These genes were subjected to quantitative real-time PCR (qRT-PCR) analysis. Primer sequences for the eight candidate genes were designed using Oligo 6.0 software, based on sequences available in the NCBI database. Details of the eight candidate genes and their corresponding primers are provided in Table 2. For the qRT-PCR analysis, we used the SYBR Premix Ex TaqTM II kit (RR820A, Takara, Dalian, China) on an Applied Biosystems 7500 Fast Real-Time PCR System (7500, ABI, Waltham, MA, USA). The qRT-PCR reaction mixture consisted of 1 µL of cDNA template, 10 µL of SYBR Premix Ex Taq, 0.4 µL of Rox Reference Dye (II), 7.4 µL of nuclease-free water, and 0.6 µL of each gene-specific primer. The amplification protocol involved an initial denaturation at 95 °C for 2 min, followed by 40 cycles of denaturation at 95 °C for 30 s, annealing at 60 °C for 30 s, and extension at 72 °C for 30 s. A melting curve analysis was then performed to confirm the specificity of the amplification. The relative expression levels of the candidate genes were quantified using the threshold cycle value (Ct) and normalized to the glyceraldehyde-3-phosphate dehydrogenase (GAPDH) gene. Expression fold changes were calculated using the ΔΔCt method, where ΔΔCt = Ct(target gene) − Ct(GAPDH).

### 2.7. Fluorescence In Situ Hybridization (FISH)

Samples of the hypothalamus were extracted from the fixative, and the target tissue was smoothed with a scalpel in a fume hood. The excised target tissue samples were dehydrated using a gradient alcohol, followed by xylene treatment for transparency. Subsequently, the samples underwent wax infiltration and paraffin embedding. The tissue blocks were then sliced into 4 µm thick sections, processed by a spreading machine, and baked in an oven at 62 °C for 2 h. The dewaxed sections were placed in DEPC-treated water and repaired in a citric acid buffer solution at pH 6.0 for 15 min. Subsequently, they were digested with 20 µg/mL proteinase K at 40 °C for 15 min, followed by three washes with PBS for 5 min each. The sections were incubated with pre-hybridization solution at 37 °C for 1 h. After the pre-hybridization solution was removed, hybridization solution containing probes (5′-GGTACACCTTGATGGGTCTCCTCTTG-3′) was added, and the sections were hybridized overnight in a constant temperature incubator. After the hybridization solution was washed away, a preheated branch-specific probe (5′-GGCGTTTTTGAACAGAGTCACCAGC-3′) hybridization solution was added, and the sections were hybridized at 40 °C for 45 min in a humid chamber. Finally, hybridization solution containing signal probes (5′-AATGGCTCATCACGTACTTGCGGA-3′) was added at 42 °C for 3 h, and the sections were incubated in the dark with DAPI dye for 8 min. After washing, the slides were sealed with an anti-fade mounting medium, with a mixture of PVA, DABCO, and DAPI in a high concentration of glycerin (Wuhan Servicebio Technology Co., Ltd., Wuhan, China), to prevent fluorescence quenching. The sections were examined under a Nikon Eclipse E100 microscope (Nikon, Tokyo, Japan) and images were captured using the NIKONDS-U3 imaging system (Nikon, Tokyo, Japan).

### 2.8. Statistical Analysis

The levels of serum biochemical indicators and the data on candidate gene expression were organized and analyzed using Microsoft Excel 2007. Statistical analyses were conducted using SPSS 26.0 software. The normality of the data distribution was assessed using the Shapiro–Wilk test, while the homogeneity of variances for all normally distributed variables was evaluated using the Levene’s test. A one-way ANOVA was performed, followed by Duncan’s multiple comparison test for post-hoc analysis. Significance levels were set at *p* < 0.05 for significant differences, *p* < 0.01 for highly significant differences, and *p* > 0.05 for nonsignificant differences.

## 3. Results

### 3.1. Blood Biochemical Indicators in Response to Fasting and Refeeding

**Figure 1:**
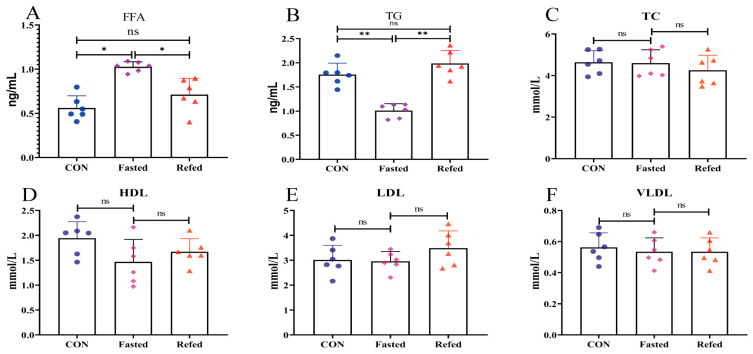
Blood lipid marker concentrations of geese under fasting and refeeding conditions. (Serum free fatty acids (FFA) (**A**), triglyceride (TG) (**B**), total cholesterol (TC) (**C**), high-density lipoprotein (HDL) (**D**), low-density lipoprotein (LDL) (**E**), and very-low-density lipoprotein (VLDL) (**F**); CON: control group; Fasted: 24 h fasting; and Refed: 24 h of fasting followed by 3 h of refeeding; *n* = 6; ns > 0.05, * *p* < 0.05, and ** *p* < 0.01).

**Figure 2:**
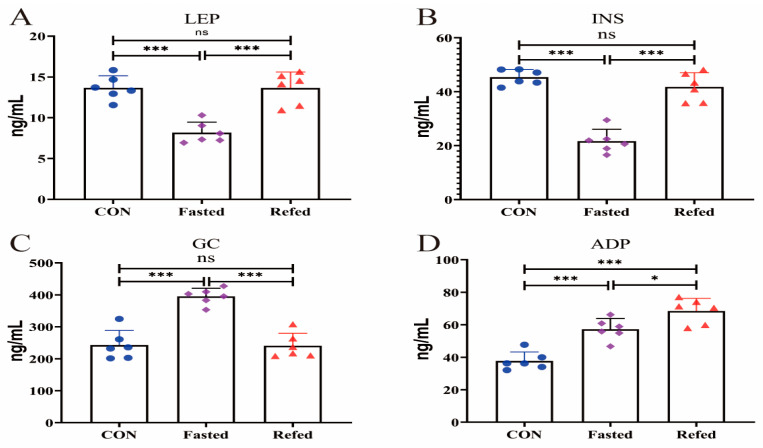
Serum appetite-related factor concentrations of geese under fasting and refeeding conditions. (Serum leptin (LEP) (**A**), insulin (INS) (**B**), glucagon (GC) (**C**), and adiponectin (ADP) (**D**); CON: control group; Fasted: 24 h fasting; and Refed: 24 h of fasting followed by 3 h of refeeding; *n* = 6; ns > 0.05, * *p* < 0.05, and *** *p* < 0.001).

Figure 1 depicts the serum levels of FFA, TG, TC, HDL, LDL, and VLDL in geese during fasting and refeeding. The concentration of serum FFA increased significantly during the fasting period but returned to pre-feeding levels after refeeding (Figure 1A). Serum TG concentrations decreased significantly during fasting and returned to pre-fasting levels after refeeding (Figure 1B). However, serum levels of TC, HDL, LDL, and VLDL remained constant during the fasting and refeeding periods (Figure 1C–F). Furthermore, Figure 2 summarizes the serum concentrations of appetite-related factors, such as insulin, glucagon, leptin, and adiponectin, during fasting and refeeding. Serum leptin and insulin concentrations exhibited a significant decrease following the fasting period but returned to pre-fasting levels upon refeeding (Figure 2A,B). Conversely, serum glucagon concentrations showed a notable increase after fasting but reverted to pre-fasting levels after refeeding (Figure 2C). Interestingly, serum adiponectin concentrations continued to progressively rise throughout both the fasting and refeeding phases (Figure 2D). These findings indicate that the significant fluctuations in energy and nutrition resulting from fasting and refeeding impact the blood lipid profile and appetite regulation in geese.

### 3.2. Analysis of DGE in Goose Hypothalamus and Subcutaneous Adipose Tissue

**Figure 3:**
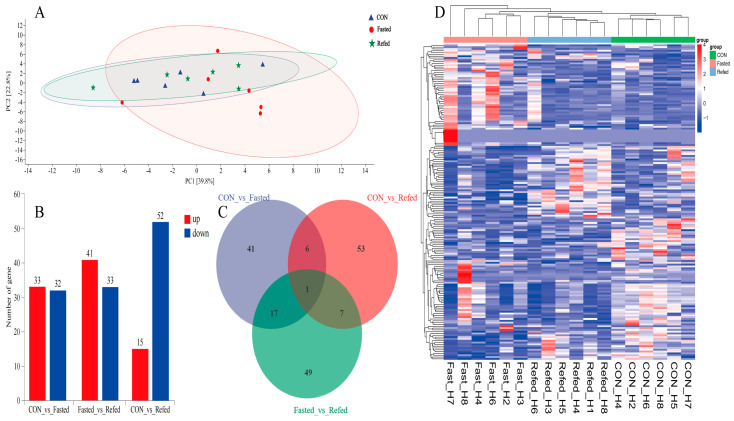
Overview of transcriptome sequencing of the goose hypothalamus, including (**A**) a principal component analysis (PCA) conducted for each mRNA−Seq sample, (**B**) the identification of differentially upregulated and downregulated genes in each group, (**C**) a Venn diagram showing the intersection for the differentially expressed genes between groups, and (**D**) a hierarchical clustering analysis of differential gene expression (DGE), with higher expression levels represented by shades of red and lower expression levels depicted in shades of steel blue. CON: control group; Fasted: 24 h fasting; Refed: 24 h of fasting followed by 3 h of refeeding; *n* = 6.

**Figure 4:**
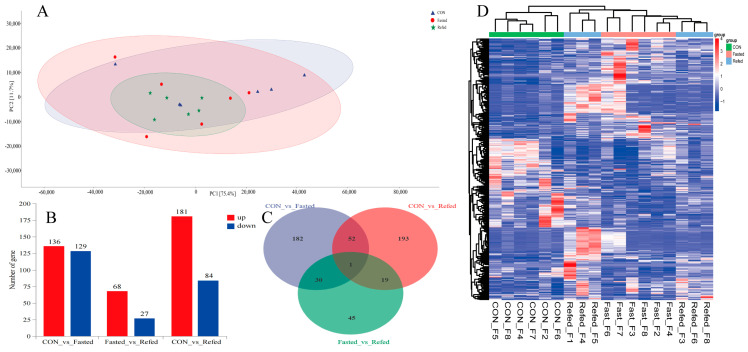
Overview of transcriptome sequencing of the goose subcutaneous fatty tissue, including (**A**) a principal component analysis (PCA) conducted for each mRNA−Seq sample, (**B**) the identification of differentially upregulated and downregulated genes in each group, (**C**) a Venn diagram showing the intersection for the differentially expressed genes between groups, and (**D**) a hierarchical clustering analysis of differential gene expression (DGE), with higher expression levels represented by shades of red and lower expression levels depicted in shades of steel blue. CON: control group; Fasted: 24 h fasting; Refed: 24 h of fasting followed by 3 h of refeeding; *n* = 6.

To investigate the effects of fasting and refeeding on the transcription profiles of the hypothalamus and subcutaneous adipose tissue in geese, we performed an RNA-Seq analysis on samples from three experimental groups, control (CON), fasted, and refed (*n* = 6), for both tissue types. The results of principal component analysis (PCA) revealed no significant differences between the groups in the hypothalamus and subcutaneous adipose tissues, indicating a cohesive clustering pattern (Figure 3A and Figure 4A). To investigate the similarities and relationships between the various libraries in the hypothalamus and subcutaneous adipose tissue, we performed hierarchical clustering analysis of differentially expressed genes (DEGs). The clustering heat map shows a consistent and significant clustering pattern among the biological replicates of each experimental group in the hypothalamus (Figure 3D). Similarly, distinct clustering patterns were seen in subcutaneous adipose tissue for the CON and fasted experimental groups, with minimal separation in the refed group (Figure 4D). In the hypothalamus, we identified a total of 65 DEGs, with 33 genes upregulated and 32 genes downregulated in the fasting group compared to the control group (Figure 3B). When we compared the refed and fasting groups, we found 74 DEGs, with 41 genes upregulated and 33 genes downregulated in the refed group (Figure 3B). Additionally, between the control and refed groups, 67 DEGs were identified, with 15 genes upregulated and 52 genes downregulated in the refed group compared to the control group (Figure 3B). We visualized the comparison of DEGs between groups using a Venn diagram (Figure 3C), revealing eighteen DEGs overlapping in both the CON vs. fasted and fasted vs. refed comparisons while seven DEGs showed an overlap in the CON vs. fasted and CON vs. refed comparisons. Additionally, eight DEGs overlapped in the fasted vs. refed and CON vs. refed comparisons, and one common gene was identified across all three comparisons (Figure 3C). In subcutaneous adipose tissues, we identified a total of 522 DEGs. Among them, 136 genes were upregulated and 129 genes were downregulated in the fasting group compared to the control group (Figure 4B). When comparing the refed group to the fasting group, 95 DEGs were identified, with 68 genes upregulated and 27 genes downregulated in the refed group (Figure 4B). Additionally, there were 275 DEGs identified between the control group and the refed group, with 181 genes upregulated and 84 genes downregulated in the refed group when compared to the control group (Figure 4B). The Venn diagram for subcutaneous adipose tissue showed that 31 DEGs overlapped in the CON vs. fasted and fasted vs. refed comparisons, while 53 DEGs overlapped in the CON vs. fasted and CON vs. refed comparisons (Figure 4C). Furthermore, 20 DEGs overlapped in the fasted vs. refed and CON vs. refed comparisons, with one common gene identified across all three comparisons (Figure 4C).

### 3.3. GO and KEGG Enrichment Analysis for DEGs

**Figure 5:**
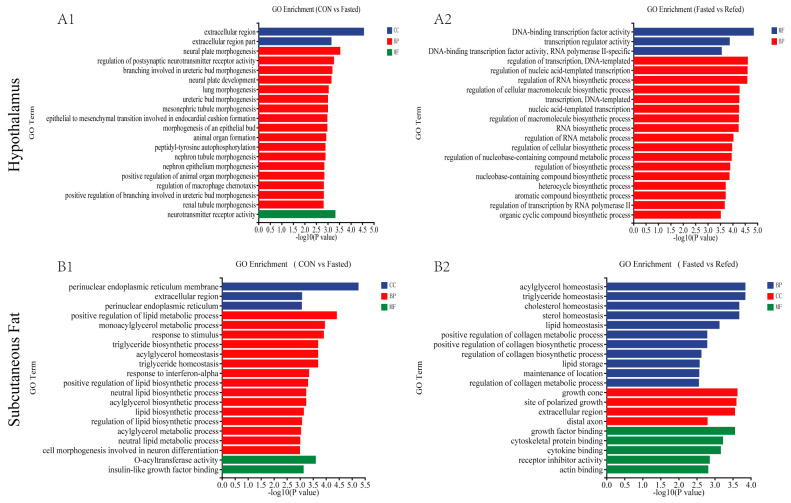
Analysis of GO enrichment in DEGs in the hypothalamus and subcutaneous adipose tissue of geese under fasting and refeeding conditions. The GO annotation terms are divided into three main categories: biological processes (BPs), cellular components (CCs), and molecular functions (MFs). The GO classification map of the hypothalamus was generated to compare CON and Fasted (**A1**), as well as Fasted and Refed (**A2**). Additionally, the GO classification map of subcutaneous adipose tissue was generated for comparing CON and Fasted (**B1**), along with Fasted and Refed (**B2**). (CON: control group; Fasted: 24 h fasting; Refed: 24 h of fasting followed by 3 h of refeeding; *n* = 6).

**Figure 6:**
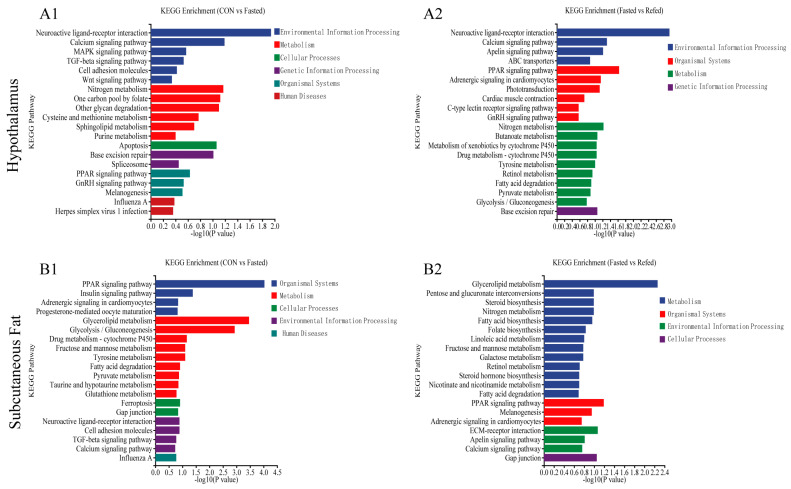
Top 20 enriched KEGG pathways of DEGs in the hypothalamus and subcutaneous adipose tissue of geese under fasting and refeeding conditions. Enriched KEGG pathways of the hypothalamus were generated to compare CON and Fasted (**A1**), as well as Fasted and Refed (**A2**). Additionally, enriched KEGG pathways of subcutaneous adipose tissue were generated for comparing CON and Fasted (**B1**), along with Fasted and Refed (**B2**). (CON: control group; Fasted: 24 h fasting; Refed: 24 h of fasting followed by 3 h of refeeding; *n* = 6).

To investigate the relationship between treatments (fasting and refeeding) and gene expression, we extracted DEGs and used gene set enrichment analysis. The analysis aimed to identify significantly overrepresented GO terms and KEGG pathways within the DEGs. The DEGs were categorized into three fundamental aspects in the GO analysis: molecular functions (MFs), cellular components (CCs), and biological processes (BPs) (Figure 5). DGEs in the hypothalamus differed primarily between the control and fasted groups in terms of extracellular region, neural plate morphogenesis, and neurotransmitter receptor activity (Figure 5A1). On the other hand, the DEGs between the fasted group and the refed group mainly associated with DNA-binding transcription factor activity, the regulation of transcription (DNA-template), and the regulation of nucleic acid-templated transcription (Figure 5A2). In subcutaneous adipose tissue, the DEGs between the control group and the fasted group primarily exhibited involvement in the perinuclear endoplasmic reticulum membrane, the positive regulation of lipid metabolic process, and O-acyltransferase activity (Figure 5B1). Meanwhile, the DEGs between the fasted group and the refed group predominantly showed associations with acylglycerol homeostasis, triglyceride homeostasis, and growth cone (Figure 5B2). Furthermore, we performed KEGG pathway enrichment analysis on the set of DEGs identified through transcriptome sequencing in the hypothalamus and subcutaneous adipose tissue. The top 20 enriched KEGG pathways for each comparison are shown in Figure 6. In the hypothalamus, the KEGG enrichment analysis revealed that the Neuroactive ligand–receptor interaction, Nitrogen metabolism, and the Calcium signaling pathway were the three most significantly enriched pathways when comparing the control group and the fasted group (Figure 6A1). When comparing the fasted group to the refed group, the pathways with the highest enrichment levels were Neuroactive ligand–receptor interaction, the PPAR signaling pathway, and the Calcium signaling pathway (Figure 6A2). When comparing the control and fasted groups in subcutaneous adipose tissue, KEGG enrichment analysis revealed that the PPAR signaling pathway, Glycerolipid metabolism, and Glycolysis/Gluconeogenesis were the three most significantly enriched pathways (Figure 6B1). When comparing the fasted and refed groups, the pathways with the highest enrichment levels were glycerolipid metabolism, the PPAR signaling pathway, and extracellular matrix (ECM) receptor interaction (Figure 6B2). Notably, the analysis of GO terms and KEGG pathways in the hypothalamus during fasting and refeeding revealed DEGs associated with a variety of pathways related to feed intake and energy metabolism. These pathways are the PPAR signaling pathway, the Apelin signaling pathway, Glycolysis/Gluconeogenesis, the Insulin signaling pathway, and the mechanistic/mammalian target of rapamycin (mTOR) signaling pathway. In contrast, the analysis of GO and KEGG pathways in subcutaneous adipose tissue indicated significant responsiveness to short-term fluctuations in nutrition and energy levels, with a predominant involvement of genes related to lipid metabolism and carbohydrate metabolism. Specifically, the PPAR signaling pathway, glycophane metabolism, fatty acid biosynthesis, and the insulin signaling pathway were well represented.

### 3.4. Identification of Key Regulatory Genes in the Hypothalamus for Short-Term Fluctuations in Nutrition and Energy Levels 

**Figure 7:**
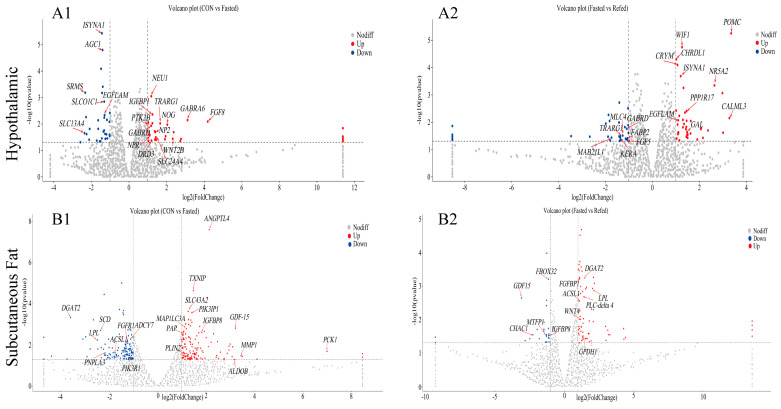
Volcano plot of DEGs in the hypothalamus and subcutaneous adipose tissue of geese under fasting and refeeding conditions. Upregulated and downregulated DEGs are shown as red and blue dots, respectively. Genes are marked with red arrows and abbreviations. The DEGs of the hypothalamus were generated to compare CON and Fasted (**A1**), as well as Fasted and Refed (**A2**). Additionally, the DEGs of the subcutaneous adipose tissue were generated for comparing CON and Fasted (**B1**), along with Fasted and Refed (**B2**). (CON: control group; Fasted: 24 h fasting; Refed: 24 h of fasting followed by 3 h of refeeding; *n* = 6).

Our research aimed to pinpoint key regulatory genes that control the hypothalamic response to short-term fluctuations in nutrient and energy levels. This investigation focused on how the hypothalamus modulates homeostatic behaviors such as food intake, energy expenditure, nutrient metabolism, and neurological processes regulation. Through an analysis of the goose RNA-seq database, we discovered 19 genes with altered expression in the hypothalamus of fasted geese compared to control geese (Figure 7A1 and  Supplementary Table S1). Thirteen of these genes are upregulated (*NOG*, *FGF8*, *WNT2B*, *DRD3*, *PTK2B*, *GABRD*, *IGFBP1*, *NP2*, *NPR*, *SLC24A4*, *TRARG1*, *GABRA6*, and *NEU1*), and they are linked to the gamma-aminobutyric acid signaling pathway, regulation of neurological system processes, glycolipid metabolic processes, and response to growth factors. The remaining six genes are downregulated (*ISYNA1*, *AGC1*, *SRMS*, *SLCO1C1*, *SLC13A4*, and *EGFLAM*), and are involved in lipid biosynthetic processes, the regulation of thyroid hormone generation, and glycoprotein metabolic processes.

**Figure 8:**
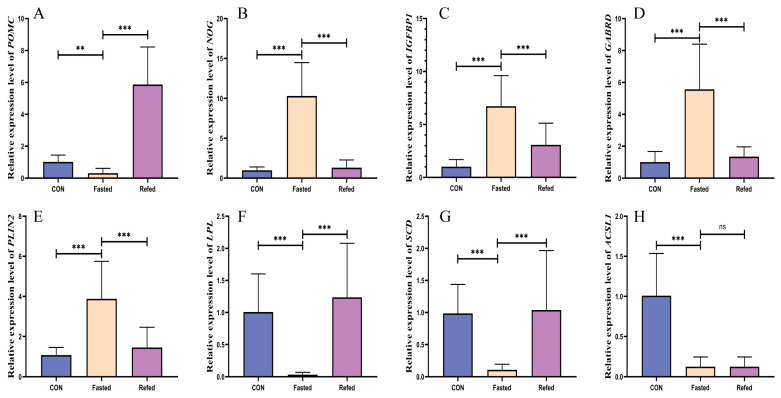
The expression of genes in the hypothalamus and subcutaneous adipose tissue of geese under fasting and refeeding conditions. (**A**–**D**) represent the expression trends of genes (POMC, NOG, IGFBP1, and GABRD) in the hypothalamus under fasting and refeeding conditions. (**E**–**H**) represent the expression trends of genes (POMC, NOG, IGFBP1, and GABRD) in subcutaneous adipose tissue under fasting and refeeding conditions. (CON: control group; Fasted: 24 h fasting; Refed: 24 h of fasting followed by 3 h of refeeding; *n* = 6; ns: *p* > 0.05, ** *p* < 0.01, and *** *p* < 0.001).

Furthermore, when we compared the fasting group and refeeding groups, we found 17 genes with altered expression in the hypothalamus (Figure 7A2 and  Supplementary Table S2). Among these genes, ten are upregulated (*POMC*, *WIF1*, *CHRDL1*, *CRYM*, *ISYNA1*, *NR5A1*, *PPP1R17*, *SRMS*, *EGFLAM*, and *GAL*), and seven are downregulated (*KERA*, *MAB21L1*, *TRARG1*, *GABRD*, *FGF5*, *FABP2*, and *MLC4*). These genes are crucial for glucose and lipid metabolism, hormonal regulation, protein metabolic processes, and the development of the visual system. To validate the accuracy of differentially expressed mRNAs identified through transcriptome sequencing technology and to investigate gene expression associated with feeding and energy regulation in response to transient changes in nutrient and energy levels, we selected four key genes for qRT-PCR analysis. These genes are *POMC*, *NOG*, *GABRD*, and *IGFBP-1* (Figure 8A–D). The qRT-PCR results demonstrate a significant upregulation in the mRNA expression of *NOG*, *GABRD*, and *IGFBP-1* genes in the hypothalamus of geese following a 24 h fasting period. Expression levels of these genes returned to baseline within 3 h after refeeding. Additionally, the *POMC* gene shows significantly lower expression in the hypothalamus of fasted geese compared to controls, with refeeding significantly increasing its expression levels. The expression patterns detected by qRT-PCR were consistent with the transcriptome analysis, confirming the reliability of our findings.

### 3.5. Identification of Key Regulatory Genes for Short-Term Fluctuations in Nutrition and Energy Levels in Subcutaneous Adipose Tissue

Our study aimed to identify key regulatory genes involved in the response of subcutaneous adipose tissue to short-term changes in nutrient and energy levels. Specifically, we focused on analyzing the regulation of lipid synthesis and breakdown within this tissue, targeting three signaling pathways: the PPAR signaling pathway, the insulin signaling pathway, and the Apelin signaling pathway. Using the goose RNA-seq database, we found 20 genes with altered expression in the subcutaneous adipose tissue of fasted geese compared to control geese (Figure 7B1 and  Supplementary Table S3). Among these genes, 12 were found to be upregulated (*PCK1*, *PLIN2*, *MMP1*, *PAP*, *ALDOB*, *MAP1LC3A*, *IGFBP8*, *ANGPTL4*, *TXNIP*, *SLC43A2*, *PIK3IP1*, and *GDF15*), and they are associated with the PPAR signaling pathway, the Apelin signaling pathway, and lipid metabolism. Furthermore, eight genes were downregulated (*SCD*, *LPL*, *ACSL1*, *PIK3R1*, *DGAT2*, *PNPLA3*, *ADCY7*, and *FGFBP1*), which participate in the PPAR signaling pathway, insulin signaling pathway, and Apelin signaling pathway. Furthermore, when comparing the fasting group to the refeeding group, we identified 12 genes with altered expression in the subcutaneous adipose tissue (Figure 7B2 and  Supplementary Table S4). Out of these genes, seven (*LPL*, *ACSL1*, *DGAT2*, *PLC-delta 4*, *GPDH1*, *WNT4*, and *FGFBP1*) were found to be upregulated, associated with the PPAR signaling pathway, Adipocytokine signaling pathway, and lipid metabolism. In addition, five genes (*IGFBP8*, *GDF15*, *FBOX32*, *MTFP1*, and *CHAC1*) were downregulated, all of which play important roles in the Apelin signaling pathway, the gamma-aminobutyric acid signaling pathway, and steroid hormone responses. To validate the accuracy of differentially expressed mRNAs identified using transcriptome sequencing technology and investigate gene expression related to lipid synthesis and breakdown in response to temporary fluctuations in nutrient and energy levels, we chose six pivotal genes for qRT-PCR analysis. These genes were lipoprotein lipase (*LPL*), acyl-CoA synthetase long chain family member 1 (*ACSL1*), stearoyl-CoA desaturase (*SCD*), and perilipin 2 (*PLIN2*) (Figure 8E–H). The analysis demonstrated a significant downregulation in the mRNA expression of *LPL*, *ACSL1*, and *SCD* genes within the subcutaneous adipose tissue of geese following a 24 h fasting period (*p* < 0.001). Additionally, except for the expression level of the *ACSL1* gene, which remained relatively low, the expression levels of *LPL* and *SCD* genes returned to baseline levels within 3 h after refeeding. Moreover, the expression of the PLIN2 gene in the subcutaneous adipose tissue of geese subjected to a 24 h fasting period was significantly higher compared to control geese (*p* < 0.05), and its expression levels decreased significantly after refeeding. The expression levels of *LPL*, *ACSL1*, *SCD*, and *PLIN2* genes in the subcutaneous adipose tissue of geese were evaluated using qRT-PCR, and the results were consistent with the transcriptome analysis.

### 3.6. Expression of the POMC Gene in the Hypothalamus during Periods of Fasting and Refeeding

**Figure 9:**
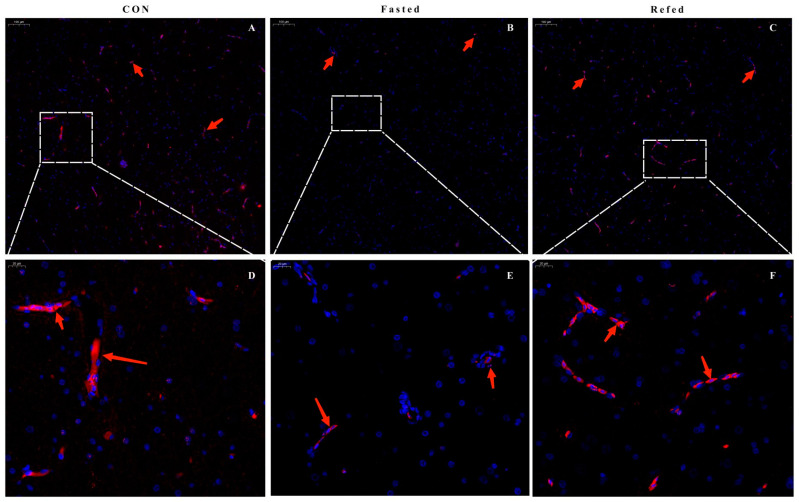
FISH for *POMC* and relative *POMC* expression in the ARC of goose hypothalamus during periods of fasting and refeeding. (**A**–**C**) Representative images of FISH for the *POMC* gene are shown, with a scale bar of 100 μm. The localization of *POMC* (red) is indicated by red arrows. (**D**–**F**) The higher magnification images of *POMC* (indicated by the white dotted box) obtained through FISH are presented, with scale bars measuring 20 μm. The localization of *POMC* (red) is indicated by the red arrows.

The *POMC* gene plays a vital role in the central nervous system, especially in regulating energy homeostasis and food intake. Our transcriptome analysis revealed significant changes in *POMC* gene expression levels in the hypothalamus between fasting and refeeding. To further investigate the relationship between *POMC* gene expression and short-term fluctuations in nutrient and energy levels, we used qRT-PCR and FISH techniques. These methods enabled us to precisely detect *POMC* gene expression changes during fasting and refeeding. Our findings revealed a significant decrease in *POMC* gene expression (represented by red arrows) in the hypothalamus of geese subjected to 24 h fasting when compared to control geese. Conversely, refeeding resulted in a significant increase in *POMC* gene expression (Figure 9). These findings are consistent with those obtained from the qRT-PCR analysis (Figure 8A).

## 4. Discussion

Fasting and refeeding systems are known to induce substantial changes in nutrient and energy levels, eliciting various physiological responses in animals. Our study sought to delve deeper into this phenomenon by analyzing the transcriptional profiles of the hypothalamus and subcutaneous fat, as well as the shifts in blood lipid levels and appetite-related factors during fasting and refeeding periods in geese. The primary objective was to enhance our understanding of how alterations in nutrition and energy affect feeding capacity and the regulation of hunger and satiety, while also shedding light on the molecular mechanisms underlying lipid metabolism in geese.

The blood biochemical indices changed significantly during fasting and refeeding, indicating the profound impact of energy and nutrition fluctuations on lipid metabolism and appetite regulation in geese. Notably, fasting triggered a marked elevation in serum FFA concentrations in geese, which reverted to pre-fasting levels upon refeeding (Figure 1A). Similar findings were reported in quail, where plasma non-esterified fatty acids (NEFAs) increased during fasting and returned to baseline within 1 h of refeeding [21]. Conversely, in chickens, serum FFA concentrations initially increased after 4 h of fasting and increased further after 8 h [34]. This fluctuation could be attributed to increased fatty acid oxidation in adipose tissue caused by fasting, which results in elevated plasma FFA levels [35]. Plasma FFAs are thought to be indirect indicators of adipose tissue lipolysis, with the majority coming from the process itself [36]. Overall, these findings indicate that short-term fasting effectively stimulates lipolysis.

This surge in lipolysis is primarily due to a consistent rise in circulating glucagon levels, which act as the primary regulator of lipolysis in birds [37]. In broilers, fasting causes a gradual increase in plasma glucagon concentrations after 6 h, followed by a decrease in plasma insulin levels [38]. Similarly, our study revealed a significant increase in serum glucagon concentration during fasting, accompanied by a decrease in serum insulin levels (Figure 2B,C). The effectiveness of the glucose–glucagon and glucose–insulin feedback mechanisms in regulating changes in plasma glucose levels in geese has been well documented [39]. Previous research has shown that birds rely on glucose mobilization to keep blood sugar levels stable during fasting, and serum glucagon increases glucose mobilization by 50%. Additionally, our findings revealed that lipid markers such as TC, HDL, LDL, and VLDL did not show significant changes during both fasting and refeeding periods. Notably, the serum concentration of leptin, a major regulator of appetite, significantly decreased post-fasting, potentially attributable to diminished hunger. Our results are consistent with previous studies, showing that most fasting-induced changes in metabolite and hormone serum levels quickly revert upon refeeding in mammals [40]. Importantly, our findings show that after a 3 h refeeding interval, all altered blood biochemical levels reverted to their pre-fasting states without any exceptions. These findings, along with previous studies, suggest that short-term fasting induces appetite regulation and metabolic adjustments in the surrounding adipose tissue of geese without inducing a negative energy balance. In summary, fasting and refeeding cause adaptive hormonal and metabolic responses, with energy and nutrient metabolism primarily regulated by the complex interaction of hormones (leptin, insulin, and glucagon) and energy substrates (free fatty acids and triglycerides). However, more evidence is needed to support the mechanism underlying geese’s transition to relying on fatty acids and ketones for energy production while fasting.

Furthermore, DEG analysis in subcutaneous adipose tissue reveals a significant response to short-term fluctuations in nutrition and energy levels. This response is mainly mediated by genes associated with lipid and carbohydrate metabolism, which include pathways such as the PPAR signaling pathway, glycophane metabolism, fatty acid biosynthesis, and insulin signaling. Our findings are consistent with previous studies on fasting’s effects on the transcriptomic characteristics of human adipose tissue. Specifically, fasting triggers the downregulation of various metabolic pathways in human fat tissue, such as triglyceride and fatty acid synthesis, glycolysis, and insulin signaling. This process involves genes linked to insulin signaling, PPAR signaling, glycogen metabolism, and lipid droplet regulation [41]. In chickens, fasting upregulates the expression of genes involved in fatty acid oxidation, proteolysis, and amino acid degradation, while decreasing the expression of genes controlling fatty acid, cholesterol, and triacylglycerol synthesis [42]. According to research, fatty acid oxidation in the adipose tissue of young broilers is dynamically regulated by nutritional status; specifically, short-term fasting (3 h) increases fatty acid oxidation in adipose tissue, with this effect increasing as the fasting period extends [35]. Similarly, our transcriptome and qRT-PCR analyses revealed a significant downregulation of genes involved in fatty acid synthesis, such as *LPL*, *SCD*, and *ACSL1* in the subcutaneous adipose tissue of geese subjected to a 24 h fasting period. In contrast, the expression of the *PLIN2* gene, which plays a crucial role in promoting hormone-stimulated lipolysis and lipid droplet metabolism, was upregulated during the fasting state. Overall, this evidence suggests that subcutaneous adipose tissue responds quickly to short-term fasting by suppressing lipogenesis and increasing lipolytic mechanisms that degrade lipid droplets.

Our transcriptional analysis of the hypothalamus in geese during a fasting–refeeding perturbation reveals new information about transcriptional regulation during short-term fluctuations in nutrient and energy availability. The analysis of DEGs in the hypothalamus revealed a complex transcriptional response to fasting and refeeding, affecting multiple metabolic pathways and key genes associated with feed intake and energy metabolism, including the PPAR signaling pathway, apelin signaling pathway, glycolysis/gluconeogenesis, insulin signaling pathway, and mTOR signaling pathway. This finding suggests that the hypothalamus, an important component of the CNS, has a direct influence on both food intake and energy homeostasis. Previous research has demonstrated comparable transcriptional alterations in the hypothalamus of chickens exposed to a low-energy diet [43]. Moreover, it has been demonstrated that food deprivation elicits intricate intra- or inter-group interactions among distinct neuronal populations and individual subtypes within the mouse hypothalamus, coordinating significant fluctuations in nutritional status and energy levels [44]. Additionally, our RNA-seq and q-PCR findings revealed significant changes in the transcriptional levels of key genes involved in growth regulation *(NOG* and *IGFBP1*), nutrient sensing (*GABRD*), and feed intake regulation (POMC) in the hypothalamus during fasting and refeeding. These results implicate a potentially pivotal role of these genes in the regulation of food intake and maintenance of nutritional homeostasis. In mammals, bone morphogenetic proteins (BMPs) work together to regulate metabolic health and modulate energy intake and expenditure via the hypothalamic pathway and peripheral tissues, including adipose tissue [45]. Noggin is an extracellular BMP inhibitor that regulates lipogenesis [46]. Following a 24 h fasting period, there was a significant upregulation in the mRNA expression of the *NOG* and *IGFBP-1* genes in the hypothalamus of geese, which reverted to baseline levels within 3 h of refeeding. This finding suggests that during times of energy and nutrient deficiency, the hypothalamus upregulates the expression of *NOG* and *IGFBP-1* genes, inhibiting growth and fat synthesis, which supports our adipose tissue transcriptome analysis. Furthermore, previous studies have implicated insulin-like growth factor (IGF)-1 and its binding proteins (IGFBPs) in appetite regulation in chicks [47]. After a 12 h fasting period, mRNA levels of *IGFBP-1* and *-2* significantly increased in chicks, which then reverted upon refeeding. Our results are consistent with those findings. Additionally, the GABAergic system has been demonstrated to stimulate food consumption in both mammals and birds. GABAergic neurons in various hypothalamic feeding nuclei serve as primary or secondary responders to negative energy balance, highlighting the pivotal role of GABA in regulating food intake [48,49,50]. In the CNS, two distinct subtypes of GABA receptors, GABA-A and GABA-B, are vital in regulating feeding behavior [51]. Central GABA-A receptor stimulation enhances feeding behavior [52,53]. Our findings demonstrate a significant upregulation in the mRNA expression of the *GABRD* gene, an essential component of the GABA-A receptor, in the hypothalamus of geese after a 24 h fasting period. These levels return to normal within 3 h of refeeding. This could be associated with food intake restriction and its potential influence on foraging behavior. Recent studies further substantiate that increased GABA levels during fasting exert inhibitory effects on POMC cells, exhibiting a negative correlation between *GABA* and *POMC* expression in POMC neurons [54,55]. We also found *GABRD* gene expression in the hypothalamus of geese increased after 24 h of fasting, while *POMC* gene expression decreased. Extensive research conducted over several decades has provided significant insights into the essential role of pro-opiomelanocortin (POMC) as a key anorexigenic factor in neural circuits related to feeding, responding to various feeding behaviors and nutritional states in both mammals [56,57] and birds [17,58]. Our consistent findings from RNA-seq, FISH, and q-PCR analyses show that fasting reduces the expression of the *POMC* gene in the hypothalamus, whereas refeeding significantly increases it. These results indicate that the hypothalamic *POMC* gene responds promptly to changes in food availability through inhibition or disinhibition.

Consistent with our findings, newly hatched chicks show a notable decrease in hypothalamic *POMC* gene expression after 24 h of fasting, with a more pronounced reduction after 48 h [59]. In mammals, the hypothalamic arcuate nucleus (ARH) contains POMC neurons, which are responsible for regulating energy and glucose homeostasis by responding to various hormonal and nutritional signals [60,61]. Acute fasting and chronic food restriction reduce hypothalamic levels of α-MSH, an anorexigenic peptide encoded by the *POMC* gene, in mice and rats [62,63]. Additionally, food deprivation reduces the excitability of POMC neurons and suppresses *POMC* gene expression [64,65]. A deficiency in the *POMC* gene and decreased *POMC* expression are associated with increased food intake, ultimately leading to severe obesity [66,67]. Similarly, reductions in POMC levels are linked to heightened appetite in chickens [68], whereas increased hypothalamic POMC expression contributes to appetite suppression in geese [69]. As a result, the POMC gene is most likely involved in the hypothalamic regulation of feed intake and nutritional status in geese.

The present study aimed to investigate the effects of fasting and refeeding on blood biochemical indicators, adipose tissue histology, and transcriptional profiles in the hypothalamus and subcutaneous adipose tissue of geese. Our findings demonstrate that fasting elicits significant alterations in various biochemical markers within the bloodstream. Transcriptomic analyses reveal a complex transcriptional response in both the hypothalamus and subcutaneous adipose tissue, influencing multiple metabolic pathways and key genes involved in feed intake and energy metabolism. This study highlights the intricate biological responses to nutritional changes in geese, which may have broader implications for understanding energy balance and metabolic regulation in avian species.

## 5. Conclusions

Our findings revealed significant alterations in biochemical indicators in the bloodstream as a result of fasting. Notably, we discovered a number of key genes involved in food consumption and energy metabolism. *LPL*, *SCD*, *ACSL1*, and *PLIN2* are genes that play important roles in fatty acid synthesis and breakdown in subcutaneous adipose tissue. In addition, we identified *NOG*, *GABRD*, *IGFBP-1*, and *POMC* genes as important hypothalamic growth and feeding regulators. These findings enhance our understanding of the molecular mechanisms underlying lipid metabolism and appetite regulation during periods of nutritional fluctuation. This knowledge could be pivotal in developing strategies to manage energy balance and metabolic health in avian species.

## Figures and Tables

**Table 1:** Feed ingredients and analyzed chemical composition of geese diets (air-dry basis %).

Ingredients	Content %	
	1–28 d	28–44 d
Corn	60.30	58.80
Soybean meal	32.60	25.60
Fish meal	2.00	10.10
Soybean oil	2.00	1.50
Lys + Met	0.10	0.00
Limestone	0.00	1.00
Premix ^a^	3.00	3.00
Total	100	100
Nutritional level		
ME/(MJ/kg)	12.13	12.55
Crude protein	20.23	16.00
Crude fiber	3.07	7.00
Ca	0.55	0.68
P	0.45	0.43

^a^ One kilogram of the premix contained the following: Fe 100 mg, Cu 8 mg, Mn 120 mg, Zn 100 mg, Se 0.4 mg, Co 1.0 mg, I 0.4 mg, VA 8330 IU, VB1 2.0 mg, VB2 2.8 mg, VB6 1.2 mg, VB12 0.03 mg, VD3 1440 IU, VE 30 IU, biotin 0.2 mg, folic acid 2.0 mg, pantothenic acid 20 mg, and niacin acid 40 mg.

**Table 2:** Real-time PCR primer sequences.

Gene Name	Primer Sequences (5′–3′)	Annealing Temperature	Size of Target Fragments
NOG	F:CAACTTCTTCCACACGCACG	60 °C	219 bp
	R:AAAGACGGCCCCCGAATATG		
GABRD	F:TGAAAGCCCACATCAGTCTTAG	60 °C	95 bp
	R:AGGCTGTGTGCAGATGTAGT		
IGFBP1	F:GCAACTGCAAGATCGAGTCC	56.5 °C	215 bp
	R:GGAACATGCTCCAACATGCC		
POMC	F:CGCAAGTACGTGATGAGCCA	62.5 °C	bp
	R:CCAGCGGAAGTGCTCCAT		
LPL	R:GGACGGTGACAGGCATGTAT	65.0 °C	150 bp
	F:CCACCAGCTTAGTGTACGCA		
ACSL1	F:CGCCGTGCTCCGCTTAAATA	60 °C	204 bp
	R:CCCATCAGCGTGTTTGTTGG		
SCD	F:AGCGACATAAAGGCCGACAA	60 °C	212 bp
	R:TTGCCAAACATGTGAGCAGC		
PLIN2	F:GGTGAGCAGTGGAATGGACA	60 °C	199 bp
	R:TAAGGCTTGCTGGTAGGCAC		
GAPDH	F:GGTGGTGCTAAGCGTGTCAT	60 °C	200 bp
	R:CCCTCCACAATGCCAAAGTT		

## Data Availability

The datasets presented in this study are available in online repositories. The names of the repository/repositories and accession numbers can be found at https://www.ncbi.nlm.nih.gov/bioproject/PRJNA1149064 (accessed on 16 August 2024) and https://www.ncbi.nlm.nih.gov/bioproject/PRJNA1149112 (accessed on 16 August 2024).

## Supplementary Materials

The following supporting information can be downloaded at: https://www.mdpi.com/article/10.3390/ani14182746/s1, Table S1: The key regulatory genes governing fasting and refeeding in the hypothalamus were compared between control and fasted conditions; Table S2: The key regulatory genes governing fasting and refeeding in the hypothalamus were compared between fasted and refed conditions; Table S3: The key regulatory genes governing fasting and refeeding in the subcutaneous adipose tissue were compared between control and fasted conditions; Table S4: The key regulatory genes governing fasting and refeeding in the subcutaneous adipose tissue were compared between fasted and refed conditions.
